# Adenosine Stimulates the Migration of Human Endothelial Progenitor Cells. Role ofCXCR4 and MicroRNA-150

**DOI:** 10.1371/journal.pone.0054135

**Published:** 2013-01-09

**Authors:** Magali Rolland-Turner, Emeline Goretti, Mélanie Bousquenaud, Frédérique Léonard, Christelle Nicolas, Lu Zhang, Fatiha Maskali, Pierre-Yves Marie, Yvan Devaux, Daniel Wagner

**Affiliations:** 1 Laboratory of Cardiovascular Research, Public Research Centre – Health (CRP – Santé), Luxembourg, Luxembourg; 2 Nancyclotep Experimental Imaging Platform, Vandoeuvre-les-Nancy, France; 3 Division of Cardiology, Centre Hospitalier, Luxembourg, Luxembourg; Centro Cardiologico Monzino, Italy

## Abstract

**Background:**

Administration of endothelial progenitor cells (EPC) represents a promising option to regenerate the heart after myocardial infarction, but is limited because of low recruitment and engraftment in the myocardium. Mobilization and migration of EPC are mainly controlled by stromal cell-derived factor 1α (SDF-1α) and its receptor CXCR4. We hypothesized that adenosine, a cardioprotective molecule, may improve the recruitment of EPC to the heart.

**Methods:**

EPC were obtained from peripheral blood mononuclear cells of healthy volunteers. Expression of chemokines and their receptors was evaluated using microarrays, quantitative PCR, and flow cytometry. A Boyden chamber assay was used to assess chemotaxis. Recruitment of EPC to the infarcted heart was evaluated in rats after permanent occlusion of the left anterior descending coronary artery.

**Results:**

Microarray analysis revealed that adenosine modulates the expression of several members of the chemokine family in EPC. Among these, CXCR4 was up-regulated by adenosine, and this result was confirmed by quantitative PCR (3-fold increase, P<0.001). CXCR4 expression at the cell surface was also increased. This effect involved the A_2B_ receptor. Pretreatment of EPC with adenosine amplified their migration towards recombinant SDF-1α or conditioned medium from cardiac fibroblasts. Both effects were abolished by CXCR4 blocking antibodies. Adenosine also increased CXCR4 under ischemic conditions, and decreased miR-150 expression. Binding of miR-150 to the 3′ untranslated region of CXCR4 was verified by luciferase assay. Addition of pre-miR-150 blunted the effect of adenosine on CXCR4. Administration of adenosine to rats after induction of myocardial infarction stimulated EPC recruitment to the heart and enhanced angiogenesis.

**Conclusion:**

Adenosine increases the migration of EPC. The mechanism involves A_2B_ receptor activation, decreased expression of miR-150 and increased expression of CXCR4. These results suggest that adenosine may be used to enhance the capacity of EPC to revascularize the ischemic heart.

## Introduction

Cardiovascular diseases are a major cause of mortality and their prevalence is expected to increase substantially [1]. Cell therapy with endothelial progenitor cells (EPC) has emerged as a promising strategy to revascularize the heart after acute myocardial infarction (MI) and thereby limit left ventricular remodeling and the incidence of heart failure (HF).

Some clinical studies [2]–[4] using EPC showed promising results, but the benefit was limited, in part, by a low retention of the injected cells in the myocardium. Improving EPC recruitment to the site of injury by increasing the expression of certain cell surface receptor has the potential to improve cardiac repair. The stromal cell-derived factor-1α (SDF-1α)/CXCR4 axis is highly implicated in EPC mobilization from the bone marrow and homing to vascular lesions [5]–[8]. Impaired CXCR4 signaling reduces the revascularization capacity of EPC in patients with coronary artery disease [9]. Moreover, the administration of endothelial colony forming cells that overexpress CXCR4 resulted in a significant increase in tissue healing and capillary density in the hindlimb ischemia model [10].

MicroRNAs (miRNAs) are short oligonucleotides able to regulate gene expression. Following ischemic stress, the expression of miR-150 in bone marrow derived mononuclear cells is inhibited [11]. Knowing that CXCR4 is a target of miR-150 [11], this raises the possibility that miR-150 may be involved in the regulation of EPC recruitment to the ischemic heart. In the infarcted heart, Adenosine (Ado) is produced by dephosphorylation of adenosine tri-phosphate (ATP) in a large amount. Ado exerts its effects through interaction with cell surface G protein-coupled receptors subdivided into four subtypes: A_1_, A_2A_, A_2B_ and A_3_ receptors [12]. Cardioprotective properties of Ado have been described in the setting of reperfusion but the effect of Ado on cardiac repair has not been studied in detail. Recent studies have shown that Ado increases the adhesion of human EPC to cardiac microvascular endothelial cells [13]. We have previously reported that Ado affects several processes involved in cardiac repair, such as extracellular matrix turnover [14], [15], angiogenesis [16]–[18] and inflammation [19]–[21]. Moreover, we recently characterized the EPC response to Ado using systems-based approaches [22].

In the present study, we determined whether Ado affects the migration of EPC.

## Materials and Methods

### Materials

All materials and reagents were from Sigma (Bornem, Belgium) unless specified. Ficoll was from ICN Flow (Asse-Relegem, Belgium). The A_2B_ Ado receptor antagonist was MRS 1754 (8-[4-[((4-Cyanophenyl)carbamoylmethyl)oxy]phenyl]-1,3-di(n-propyl)xanthine). EHNA (erythro-9-(2-Hydroxy-3-nonyl) adenosine hydrochloride) was used as Ado deaminase inhibitor and dipyridamole (DIP) was used as inhibitor of Ado intracellular uptake. CADO (2-Chloroadenosine) and 8-SPT (8-(p-Sulfophenyl)theophylline hydrate) were used as non-specific agonist and antagonist of Ado receptors, respectively. The E-Toxate® reagent from *Limulus polyphemus* (LAL assay having a detection sensitivity of 0.05 EU/mL) was used to ensure the absence of endotoxin contamination in Ado and other drugs used in the study. SDF-1α was purchased from Peprotech (London, UK). Anti CXCR4 neutralizing antibodies and SDF-1α ELISA detection kit were from R&D System (Oxon, UK).

### Cell culture

Peripheral blood mononuclear cells (PBMCs) were isolated by Ficoll gradient using Leucosep tubes (Greiner Bio-One, Wemmel, Belgium). Early EPC were obtained as previously described by others [23]. Shortly, 8.10^6^ PBMCs were seeded onto human fibronectin (2.5 µg/cm^2^) pre-coated 6-well plates and cultured in EBM medium supplemented with bovin brain extract, human recombinant endothelial growth factor, hydrocortisone, gentamicin, amphotericin B and 20% fetal calf serum (FCS) (Laboratoires Eurobio, Les Ulis, France). After 3 days of culture, non-adherent cells were discarded and adherent cells were cultured for another 24 hours prior to treatment. EPC were characterized by dual positive staining for 1,1_-dioctadecyl-3,3_,3_-tetramethylindo-carbocyanine-labeled acetyl-low-density lipoprotein (DilAcLDL) (Invitrogen, Merelbeke, Belgium) and lectin from Ulex europaeus. By flow cytometry, early EPC were CD133+/CD34+/CD45+/CD14+/CD31+/vWF+/VEGFR2+/CD144-/CD105+. The cell population typically contained over 95% of CD14+/CD45+/CXCR4+ cells. Ischemic culture conditions were achieved by incubation of EPC with 0.1% BSA under 1% O_2_ and 5% CO_2_. Human primary cardiac fibroblasts purchased from TCS CellWorks Ltd were cultured in fibroblast medium supplemented with fibroblast growth supplement and 2% FCS (TCS CellWorks Ltd, Buckingham, UK). Prior to the experiments, fibroblasts were rendered quiescent in fibroblast medium supplemented with insulin (10 µg/mL), transferrin (5.5 µg/mL), sodium selenite (5 pg/mL), BSA (0.5 mg/mL) and linoleic acid (4.7 µg/mL) for 5 hours. Cells were incubated with Ado and EHNA (10 µmol/L). A_2B_ receptor antagonist was added 15 min prior to Ado treatment. Primary human monocytes were isolated from healthy volunteers, as described [14], [15].

### Flow cytometry

Cells were harvested using cell dissociation solution and washed twice in phosphate-buffered saline (PBS, Lonza, Verviers, Belgium) supplemented with 1% BSA. 200,000 cells were treated with the appropriate amount of Fc blocking reagent (Miltenyi, Utrecht, The Netherland) and were then stained with specific antibodies or the corresponding isotype control in 50 µL PBS-1% BSA for 30 min at 4°C. After washing, cells were fixed using the commercial BD FACS lysis solution according to manufacturer's instructions. All antibodies were purchased from Immunotools, except CD184-APC (BD Biosciences, Erembodegem, Belgium). To eliminate dead cells from analysis, LIVE/DEAD® Fixable Near-IR Dead Cell Stain Kit was used according to the manufacturer's instructions (Invitrogen). 20,000 to 40,000 events were acquired on a BD FACSCanto™ flow cytometer and analysis was performed with the FACSDiva^TM^ software (BD Biosciences). Overlays were obtained using FlowJo 7.2.4 software.

### RNA interference assay

Cells were transfected using INTERFERin^TM^ siRNA transfection reagent according to the manufacturer's instructions (Polyplus-transfection, Illkirch, France). Cells were transfected with 20 nM HS_ADORA2B_6_HP (Qiagen, Venlo, The Netherlands), pre-miR^TM^ miRNA precursor hsa-miR-150, anti-miR^TM^ miRNA inhibitor hsa-miR-150 or their respective negative controls (Applied Biosystems, Lennik, Belgium). INTERFERin-RNA complexes were allowed to form for 10 min at room temperature in a final volume of 100 µL of serum free medium before addition to the cells. After 24 or 48 hours, cells were treated with 10 µmol/L Ado for 4 or 6 hours prior harvesting.

### Real-time quantitative PCR

For assessment of messenger RNA expression, total RNA was isolated using TriReagent® and the RNeasy® mini kit (Qiagen) according to the manufacturer's instructions. Potential contaminating genomic DNA was digested by DNase I treatment (Qiagen). One µg of total RNA was reverse-transcribed using the Superscript® II Reverse Transcriptase (Invitrogen). PCR primers were designed using the Beacon Designer software (Premier Biosoft, Palo Alto, USA) and were chosen to encompass an intron. Primer details are shown in Table 1. PCR was performed using the iCycler® and the IQTM SYBR® Green Supermix (Bio-Rad, Nazareth Eke, Belgium). 1/10 dilution of cDNA was used. PCR conditions were as follows: 3 min at 95°C, 30 sec at 95°C and 1 min annealing (40-fold). Optimal annealing temperature was determined for each primer pair. Melting point analysis was obtained after 80 cycles for 10 sec from 55°C up to 95°C. Each run included negative reaction controls. β-actin was chosen as housekeeping gene for normalization. Expression levels were calculated by the relative quantification method (ΔΔCt) using the Genex software (Bio-Rad) which takes into account primer pair efficiency.

**Table 1:** Quantitative PCR primers.

Gene	Accession number	Forward primer	Reverse primer	T°C
β-actin	NM_001101	AGAAAATCTGGCACCACACC	GGGGTGTTGAAGGTCTCAAA	60
CXCR4	NM_001008540	TATCCTGCCTGGTATTGTC	GGAAATCATCAAGCAAGGG	50
A_1_	NM_000674	GACCTACTTCCACACCTG	TCACCACCATCTTGTACC	58
A_2A_	NM_000675	TCTTCAGTCTCCTGGCCATC	GGGACCACATCCTCAAAGAG	64
A_2B_	NM_000676	TCCATCTTCAGCCTTCTG	GCACTGTCTTTACTGTTCC	55
A_3_	NM_000677	TCATCTGCGTGGTCAAGC	CTGTAGAAGTGGATTGTGATGC	62

Annealing temperatures are indicated.

For assessment of miRNA expression, total RNA was extracted using the miRVana isolation kit (Applied Biosystems,). Five hundred ng of total RNA were used for reverse transcription using miScript reverse transcription kit (Qiagen). PCR was performed using the miScript SYBR-green PCR kit and the Homo sapiens_miR-150miScript Primer Assay (MS00003577) according to manufacturer’s instructions (Qiagen). U6 (Homo sapiens_RNU6-2_1 miScript Primer Assay, MS00033740, Qiagen) was used for normalisation. Expression levels were calculated by the ΔΔCt method.

### Microarray

Cellular mRNA was analyzed by microarray as described previously [24]. Briefly, total RNA was analyzed for quantity (Nanodrop, Nanodrop products, Wilmington, USA) and purity (Bio Analyser, Agilent Technologies, Santa-Clara, USA) before amplification using the Amino Allyl Message Amp® kit (Ambion, Austin, USA). cDNA obtained after retrotranscription was labelled with Cy3 or Cy5, and hybridized onto human microarrays covering 25,000 genes [25]. Microarrays were scanned using an Axon 3000 B scanner (Molecular Devices, Sunnyvale, USA) and raw data were acquired with Genepix software (Molecular Devices). Data are available at the Gene Expression Omnibus database, accession number GSE26744. Expression levels were compared using Significance Analysis of Microarrays software and heat maps were drown using TreeView software.

### ELISA

Concentration of SDF-1α in conditioned medium was measured using the Quantikine DSA00 ELISA kit (R&D Systems). Detection limit of the assay was 18 pg/mL.

### In vitro migration assay

Chemotaxis experiments were performed using Costar® Transwell^TM^ Permeable Supports (Corning, Amsterdam, The Netherlands) with 5 µm pore size membrane. During the whole experiment, EPC medium containing no antibiotics and 5% FCS was used. Early EPC were treated for 6 hours with 10 µM Ado/EHNA or corresponding control treatment prior harvesting using cell dissociation solution. Cells were then incubated for 1 hour at 37°C with 10 µg/mL of anti-CXCR4 neutralizing antibody or isotype control, and 175,000 cells were seeded in the upper chamber of the Transwell^TM^. Transwells^TM^ were then placed into 24-wells plate containing SDF-1α (0 to 100 ng/mL) or conditioned medium from fibroblasts. Cell migration was measured by DNA staining using CyQUANT® Cell Proliferation Assay Kit (Invitrogen, Eugene, Oregon, USA). Fluorescence was measured at 492/520 nm using a POLARstar OPTIMA (BMG Labtech, Paris, France).

### Luciferase reporter assay

Human embryonic kidney (HEK) 293T cells were used for luciferase assay and maintained in DMEM supplemented with 10%FBS, 1% L-glutamine and 1% penicillin-streptomycin. HEK-293T cells were plated into 96-well plates and co-transfected using Lipofectamine 2000 (Invitrogen) with 10 ng of a reporter plasmid containing the 3′ untranslated region (UTR) of CXCR4 inserted downstream of the Gaussian luciferase secreted reporter gene and the secreted alkaline phosphatase tracking gene (pEZX-MT05, GeneCopoeia, Labomics, Nivells, Belgium) and 30 nmol/L of pre-miR^TM^ miRNA precursor hsa-miR-150 (Applied Biosystems) or negative control. Gaussian luciferase and alkaline phosphatase activities were measured by luminescence in conditioned medium 48 hours after transfection using the secreted-pair dual luminescence kit (GeneCopoeia). Gaussian luciferase activity was normalized to alkaline phosphatase activity.

### In vivo experiments

This study was conducted in accordance with the regulations of the Animal Welfare Act of the National Institutes of Health Guide for the Care and Use of Laboratory Animals (NIH Publication No. 85–23, revised 1996). Protocols were approved by local Ethics Committee and by the Regional Veterinary Department. 23 Wistar rats underwent permanent occlusion of the left anterior descending coronary artery (LAD) to induce MI [26]–[27]. 48 hours post surgery, 18F-Fluorodeoxyglucose (FDG) Positron Emission Tomography (PET) was performed to measure infarct size and myocardial damage as defined by cardiac segments showing 18F-FDG uptake <50%. LAD-occluded animals were randomized according to infarct size into 3 groups: vehicle (NaCl, n = 7), CADO (2 mg.kg^−1^.d^−1^, n = 8) and CADO (2 mg.kg^−1^.d^−1^) with 8-SPT (10 mg.kg^−1^.d^−1^, n = 8). These treatments were given intraperitoneally twice daily for 2 months, starting 7 days after LAD occlusion. After 2 months, rats were sacrificed and hearts were harvested to perform contiguous frozen sections (8 µm), oriented along the vertical or horizontal long axis of the left ventricle depending on infarct location. Sections were fixed and permeabilized with methanol for 10 minutes, and blocked with BSA and serum for 1 hour. Polyclonal goat anti aldhehyde dehydrogenase 2 (ALDH2) (Santa Cruz Biotechnology, Heidelberg, DE), mouse monoclonal anti CD31 (clone P2B1, Abcam, Cambridge, UK) and polyclonal rabbit anti CXCR4 (Abcam) were used as primary antibodies. Alexa Fluor®488-coupled donkey anti-mouse antibody, Alexa Fluor®568-coupled donkey anti-goat antibody and Alexa Fluor®688-coupled donkey anti-rabbit antibody (Invitrogen) were used as secondary antibodies. Staining specificity was ensured by omission of primary antibodies. DAPI (blue) was used to stain nuclei. Hematoxylin and eosin staining was performed to assess vascularization in heart sections. The number of vessels was counted in the whole section of each animal. Images were recorded with a confocal microscope (Zeiss Laser Scanning Microscope LSM 510) and the LSM 510 META software.

### Statistical analysis

Results are presented as mean ± SD. Comparisons between two groups of continuous data were performed with two-tailed t-test for data with a normal distribution and Mann-Whitney test for non-normally distributed data. Normality was determined using the Shapiro-Wilk test. Comparisons between multiple groups were performed with one-way ANOVA for normal data and Kruskal-Wallis one-way ANOVA on ranks for non-normally distributed data. In case of significance, post-hoc tests (Dunn's or Holm-Sidak method) were run to isolate groups that differ from the others. Comparisons between multiple groups with two parameters were performed with two-way ANOVA. Statistical tests were performed with the SigmaPlot v11.0 software. A *P* value <0.05 was considered significant.

## Results

### Adenosine increases CXCR4 mRNA expression in EPC

**Figure 1:**
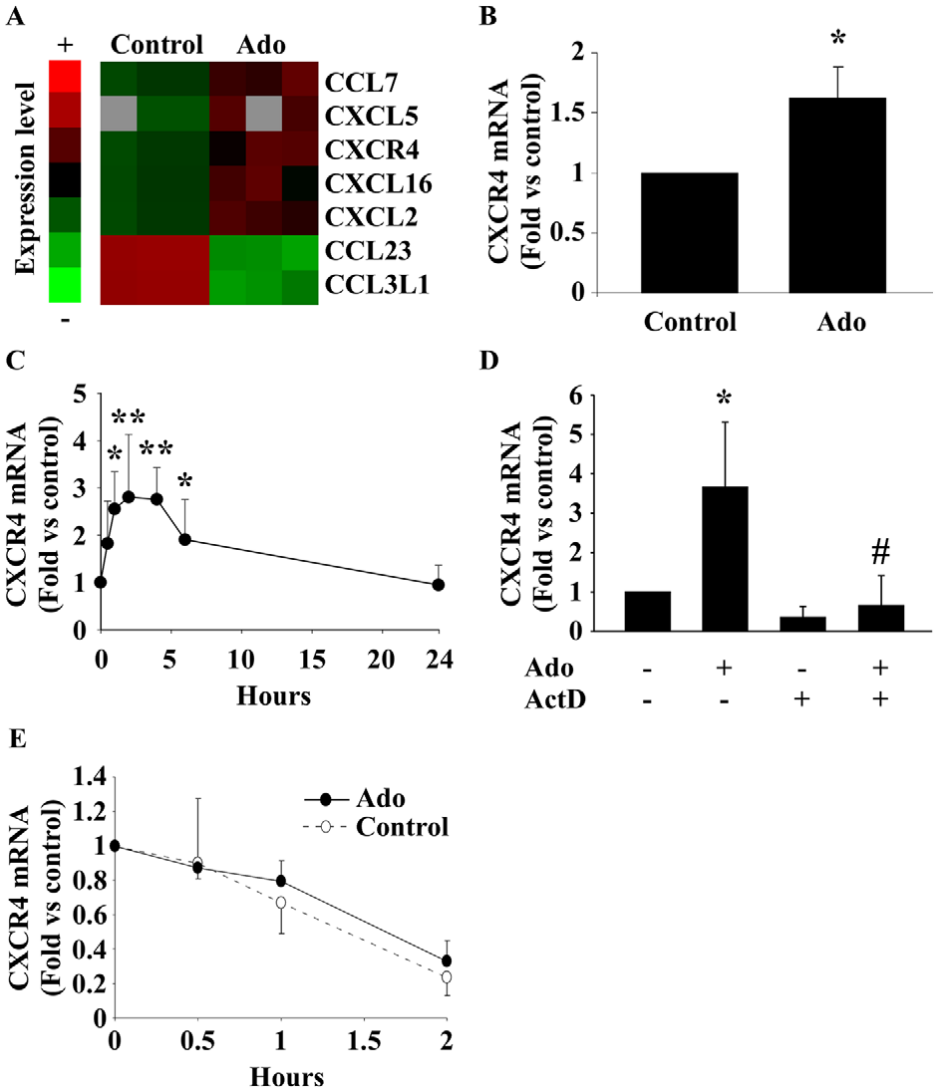
Adenosine increases CXCR4 mRNA expression. A. Transcriptomic analysis of EPC treated with 10 µmol/L Ado for 6 hours using microarrays. The heat map indicates chemokines and chemokine receptors differentially expressed following Ado treatment (all with q values ≤0.001). B. Confirmation of CXCR4 over-expression in EPC treated by 10 µmol/L Ado for 6 hours using quantitative PCR. Expression values were normalized to β-actin and to untreated cells. * P<0.05 vs control (n = 3). C. Kinetic of the effect of Ado on CXCR4 mRNA expression. * P<0.05 vs control. ** P<0.001 vs control. P<0.001 using ANOVA (n = 5). D. Effect of actinomycin D (ActD). EPC were pre-treated by 5 µg/mL ActD for 30 min before treatment with 10 µmol/L Ado. CXCR4 mRNA expression was assessed after 2 hours. * P<0.01 vs control. # P<0.01 vs Ado (n = 3). E. EPC were pre-treated with ActD (5 µg/mL) for 30 min and were incubated with Ado for different time periods (30 min, 1 h and 2 h) before assessment of CXCR4 mRNA by PCR (n = 4).

Microarray experiments were performed to investigate whether Ado modulates the expression of members of the chemokine/receptor family. These experiments showed that a 6-hour treatment of EPC with 10 µmol/L Ado modulates the expression of various chemokines and chemokine receptors. In particular, the chemokine receptor CXCR4 was up-regulated (Fig. 1A). Increased expression of CXCR4 by Ado was confirmed by PCR (Fig. 1B). Treating EPC with 10 μmol/L Ado induced a transient increase of CXCR4 mRNA expression, reaching its maximum after 2 hours (3-fold, P<0.001) and declining after 4 hours (Fig. 1C). Inhibition of de novo mRNA transcription with actinomycin D abolished this increase (Fig. 1D). Degradation over time of CXCR4 mRNA in actinomycin D pre-treated EPC was not modified by Ado treatment (P = 0.44, two-way ANOVA) suggesting that Ado does not affect CXCR4 mRNA stability (Fig. 1E). Together, these data show that Ado enhances CXCR4 mRNA expression in EPC through up-regulation of CXCR4 transcription.

Since EPC are derived from PBMCs and monocytes, we tested whether adenosine modified CXCR4 expression in primary human monocytes, No significant effect was found (data not show).

### Adenosine increases CXCR4 expression at the cell surface

**Figure 2:**
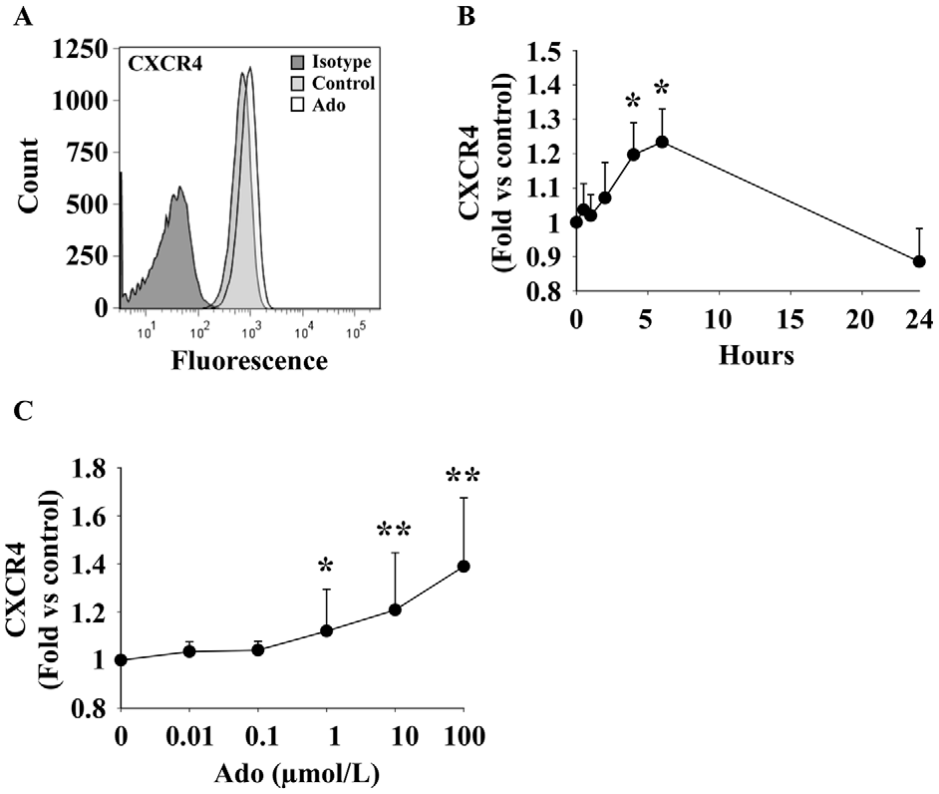
Adenosine enhances cell surface expression of CXCR4. EPC were treated with Ado and CXCR4 expression was assessed by flow cytometry. A. Representative histogram of EPC treated by 10 µmol/L Ado for 6 hours. Dark grey: isotype control. Light grey: CXCR4 staining of control cells. White: CXCR4 staining of Ado-treated cells. B. Kinetic of CXCR4 expression on EPC treated by 10 µmol/L Ado. * P<0.001 vs control (n = 10). C. Dose-dependent effect of Ado on CXCR4 expression on EPC harvested 6 hours after treatment. * P<0.05 vs control. ** P<0.01 vs control (n = 3).

Cell surface expression of CXCR4 was assessed by flow cytometry. As shown in Figure 2A, we observed a clear shift between the isotype control and the specific anti-CXCR4 antibody, indicating that EPC express CXCR4. A moderate increase of staining was noticed after Ado treatment (Fig. 2A). Time-course experiments revealed a maximal effect of Ado after 6 hours (23% increase, P<0.001) (Fig. 2B). This effect was dose-dependent and reached statistical significance at 1 µmol/L (Fig. 2C). These results show that physiological concentrations of Ado induce a moderate increase of CXCR4 expression at the surface of EPC.

### Characterization of Ado receptor expression in EPC

**Figure 3:**
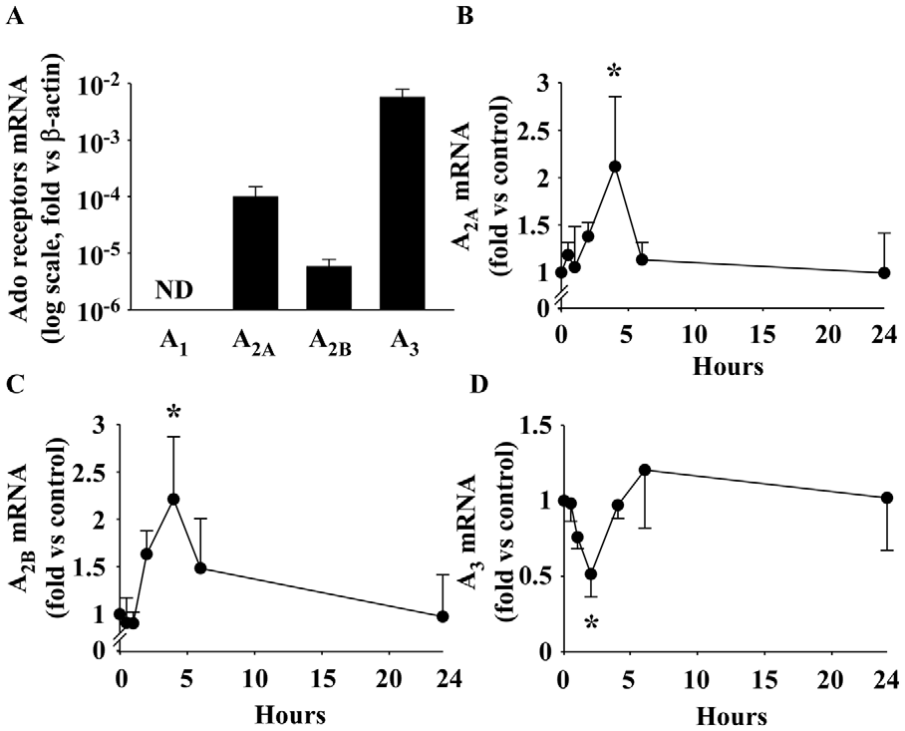
Expression of Ado receptors in EPC. A. mRNA expression profiles of the 4 Ado receptors in EPC were obtained by quantitative PCR. Significant differences were observed (n = 3, P = 0.004), the A_3_ subtype being predominant and the A_1_ subtype not detected (ND). Expression values were normalized to β-actin. B–D. Ado regulates the expression of its own receptors in EPC. mRNA expression was assessed by quantitative PCR at different times after treatment with 10 µmol/L Ado (n = 3). Ado transiently increased A_2A_ and A_2B_ expression (P = 0.003 and P = 0.002, respectively) and decreased A_3_ expression (P = 0.01). * P≤0.01 vs control.

Expression of the 4 Ado receptors in EPC was as follows: A_3_>A_2A_>A_2B_. The A_1_ receptor was not detected (Fig. 3A). Interestingly, expression of Ado receptors was regulated by Ado itself. A_2A_ and A_2B_ receptors were more than two-fold up-regulated by Ado whereas A_3_ receptor was down-regulated. Maximal effects were observed 2–4 hours after administration of Ado (Fig. 3B–D).

### The A_2B_ receptor mediates the effect of Ado on CXCR4

**Figure 4:**
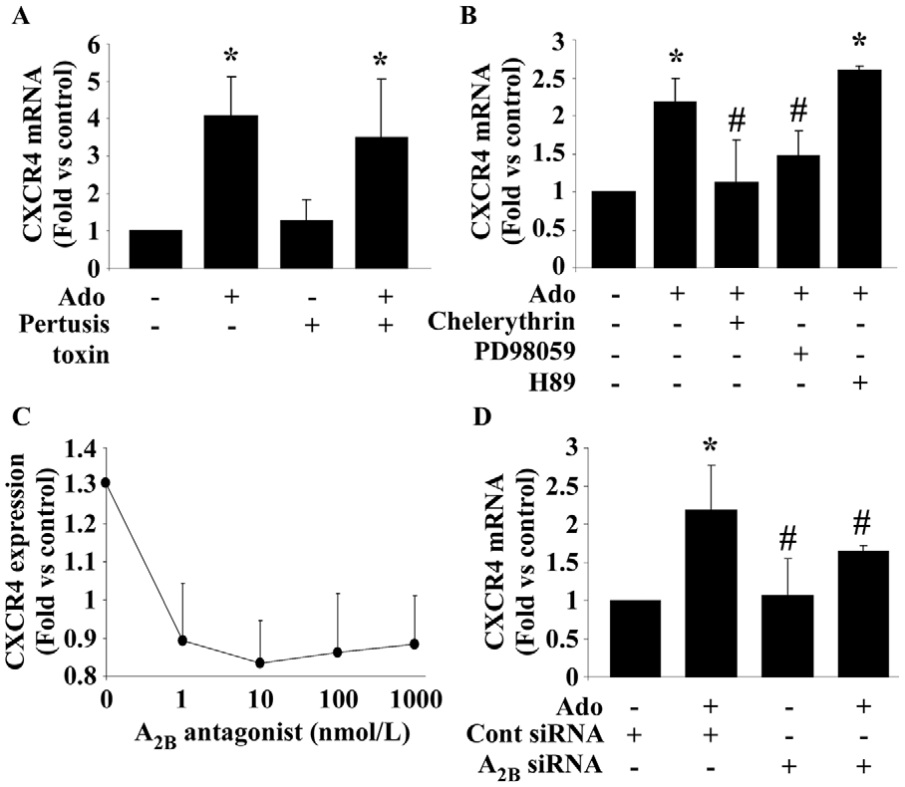
Identification of Ado receptors implicated in the effect of Ado on CXCR4. A. Pertussis toxin did not affect CXCR4 expression. Pertussis toxin (100 ng/mL) was added to the cells 30 min before Ado and CXCR4 expression was measured by PCR after 2 hours. * P<0.01 vs control (n = 5). B. Chelerythrin blocked the increase of CXCR4 expression induced by Ado. EPC were pre-treated with 10 µmol/L H89, PD98059 or chelerythrin for 15 min before 10 µmol/L Ado treatment for 4 hours. CXCR4 expression was measured by PCR. * P<0.001 vs control. # P<0.01 vs Ado (n = 3). C. A_2B_ antagonist inhibited CXCR4 over expression induced by Ado. EPC were pre-incubated for 15 min with increasing concentrations of the A_2B_ antagonist (MRS1754) or vehicle before incubation with 10 µmol/L Ado for 6 hours. CXCR4 expression was measured by flow cytometry. P = 0.02 (n = 3). D. A_2B_ gene silencing inhibited CXCR4 expression. CXCR4 mRNA expression was measured in EPC transfected with A_2B_ siRNA or control siRNA for 24 to 48 hours and treated with 10 µmol/L Ado for 4 hours. A_2B_ siRNA inhibited the increase of CXCR4 expression induced by Ado. * P<0.01 vs control. # P<0.05 vs Ado + control siRNA (n = 3).

Several approaches were used to identify the subtype of Ado receptor(s) involved in the increase of CXCR4 expression. First, inhibition of G_i_ protein signaling was induced using pertussis toxin (Fig. 4A). This inhibition did not affect the increase of CXCR4 mRNA by Ado. Since A_1_ and A_3_ receptors are coupled to G_i_ proteins, this observation excludes the implication of these 2 receptors in the effect of Ado on CXCR4. Secondly, we used pharmaceutical inhibitors of downstream signaling pathways of the A_2A_ and A_2B_ receptors, ERK and PKC, respectively (Fig. 4B). The PKC inhibitor chelerythrin prevented the increase of CXCR4 induced by Ado, suggesting the involvement of the A_2B_ receptors. The partial effect of the MEK inhibitor PD98059 suggested some contribution of the A_2A_ receptor. This contribution might involve the ERK pathway and not the PKA pathway downstream the A_2A_ receptor, since blockade of the latter pathway by H89 did not affect CXCR4 expression. Of note, none of the inhibitors used alone affected CXCR4 expression (not shown). To confirm these results, we blocked the A_2B_ receptor using the specific antagonist MRS1754 (Fig. 4C). A robust decrease of CXCR4 expression was observed. Finally, down-regulation of A_2B_ receptor using siRNA inhibited the effect of Ado on CXCR4 (Fig. 4D). A potential toxic effect of A_2B_ siRNA, which could be responsible for the lack of response of the cells to Ado, was ruled out using flow cytometry experiments (data not shown). Taken- together, these results show that activation of the A_2B_ receptor up-regulates CXCR4 expression.

### Adenosine stimulates EPC chemotaxis

**Figure 5:**
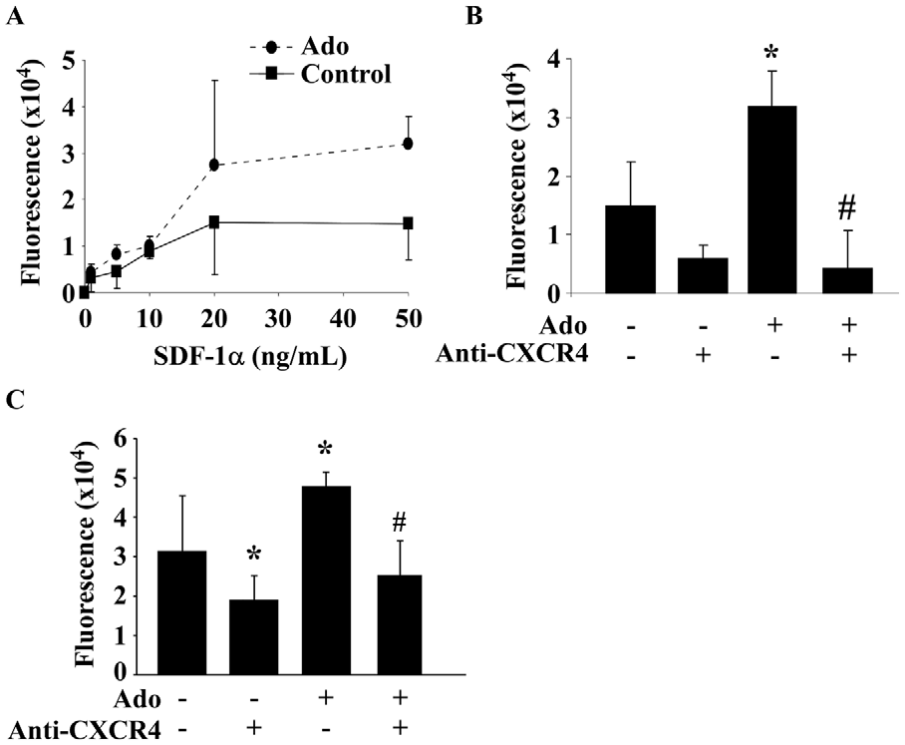
Adenosine enhances the migration of EPC. EPC were treated by 10 µmol/L Ado for 6 hours before transfer to the top of a Boyden chamber. The bottom of the chamber was filled with culture medium containing either recombinant SDF-1α (A and B) or conditioned medium from cardiac fibroblasts (C). The amount of cells that migrated through the membrane was assessed by fluorescence after 16 hours. A. SDF-1α induced EPC migration and Ado enhanced this effect (P = 0.02, two-way ANOVA. n = 3). B. Blocking CXCR4 with a neutralizing antibody (10 µg/mL) for one hour before exposure to 50 ng/mL SDF-1α for 16 hours in a Boyden chamber inhibited the migration in both untreated and Ado-treated cells. * P<0.05 vs control. # P<0.01 vs Ado (n = 3). C. Ado enhances the migration of EPC induced by conditioned medium from cardiac fibroblasts. EPC were treated by 10 µmol/L Ado or vehicle for 6 hours before transfer to the top of a Boyden chamber. The bottom of the chamber was filled with cell-free conditioned medium from fibroblasts. In some samples, EPC were pre-incubated for 1 hour with anti-CXCR4 blocking antibodies before transfer to the chambers. The amount of EPC that migrated through the membrane was assessed by fluorescence after 16 hours. * P<0.01 vs control. # P<0.001 vs Ado (n = 7).

To evaluate the effect of Ado on EPC migration, EPC were treated with 10 µmol/L Ado for 6 hours and exposed to recombinant SDF-1α (0 to 100 ng/mL) in a Boyden chamber. SDF-1α dose-dependently enhanced EPC migration and pre-treatment with Ado amplified this effect (P = 0.02; Fig. 5A). The migration was inhibited by CXCR4 blocking antibodies in both untreated and Ado-treated cells (Fig. 5B).

Cardiac fibroblasts represent a major source of SDF1α in the myocardium. We observed that treatment of EPC with 10 µmol/L Ado enhanced their migration towards a conditioned medium from fibroblasts. This increase was abolished when cells were pre-incubated with anti-CXCR4 antibodies (Fig. 5C). These data show that Ado improves the migration of EPC, at least through up-regulation of CXCR4 expression.

### Role of miR-150 in the effect of adenosine on CXCR4

**Figure 6:**
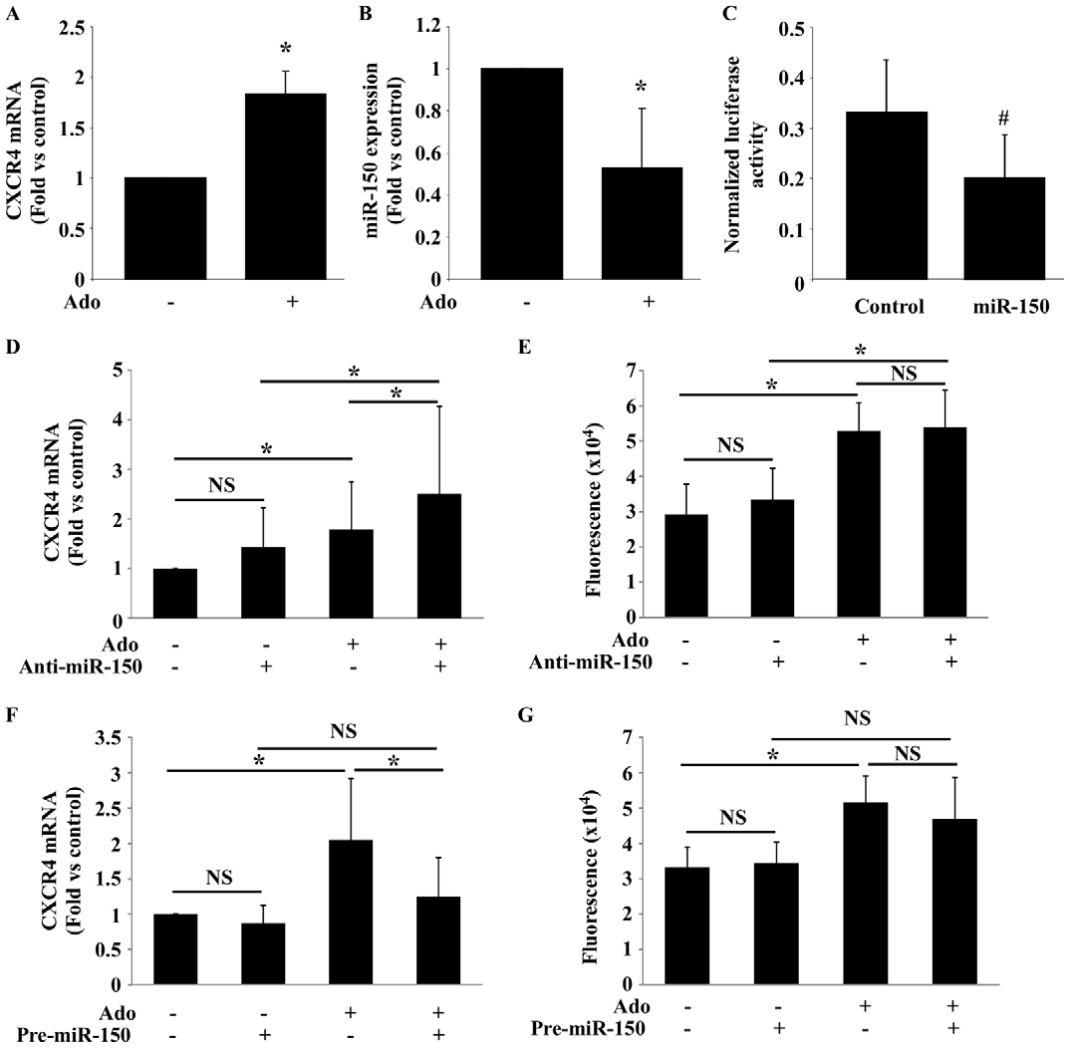
Role of miR-150 in the effect of adenosine on CXCR4. A–B. EPC were treated with 10 µmol/L Ado before incubation for 4 hours in ischemic conditions (0.1% BSA instead of 20% SVF and 1% O_2_). CXCR4 and miR-150 expression was evaluated by PCR. Results were normalized to β-actin and U6, respectively. Ado increased CXCR4 expression (A) and decreased miR-150 expression (B). * P<0.05 vs control (n = 5). C. HEK-293T cells were co-transfected with 10 ng of a reporter plasmid containing the 3′ UTR of CXCR4 and 30 nmol/L of either pre-miR control (control) or pre-miR-150 (miR-150). miR 150 decreased luciferase activity. # P<0.001 vs control. Data are from 3 independent experiments performed in triplicate. D–G. EPC were transfected with 30 nmol/L of anti-miR-150 or pre-miR-150, or respective controls, during 24 hours, and then treated with 10 µmol/L of adenosine during 6 hours in ischemic conditions. (D–F) CXCR4 expression was measured by PCR. (E–G) Cell migration towards recombinant SDF-1α was assessed in a Boyden chamber after 16 hours. * P<0.05 (n = 5). NS: not significant.

Following experiments were performed under serum starvation (0.1% BSA) and oxygen deprivation (1% O_2_). As observed in normal conditions, Ado up-regulated CXCR4 mRNA expression (Fig. 6A). This was paralleled by decreased miR-150 (Fig. 6B). A luciferase assay was used to verify the ability of miR-150 to bind CXCR4. In HEK293T cells transfected with a reported plasmid containing the 3′ UTR of the CXCR4 gene, we observed an inhibition of luciferase activity upon administration of pre-miR-150 (Fig. 6C).

Transfection of cells with anti-miR-150 to inhibit miR-150 expression had no effect on CXCR4 expression or migration towards SDF-1α (Fig. 6D–E). Associating anti-miR-150 with Ado significantly increased CXCR4 expression, compared to either treatment alone (Fig. 6D). However, anti-miR-150 did not enhance the effect of Ado on cell migration (Fig. 6E). Increasing the expression of miR-150 by addition of pre-miR-150 did not affect CXCR4 expression, but blunted the increase of CXCR4 expression induced by Ado (Fig. 6F). In migration assay, pre-miR-150 had no significant effect (Fig. 6G). These results suggest that, in EPC subjected to ischemic conditions, the increase of CXCR4 expression by Ado is, at least in part, controlled by miR-150.

### Adenosine improves EPC recruitment to the infarcted heart and angiogenesis

**Figure 7:**
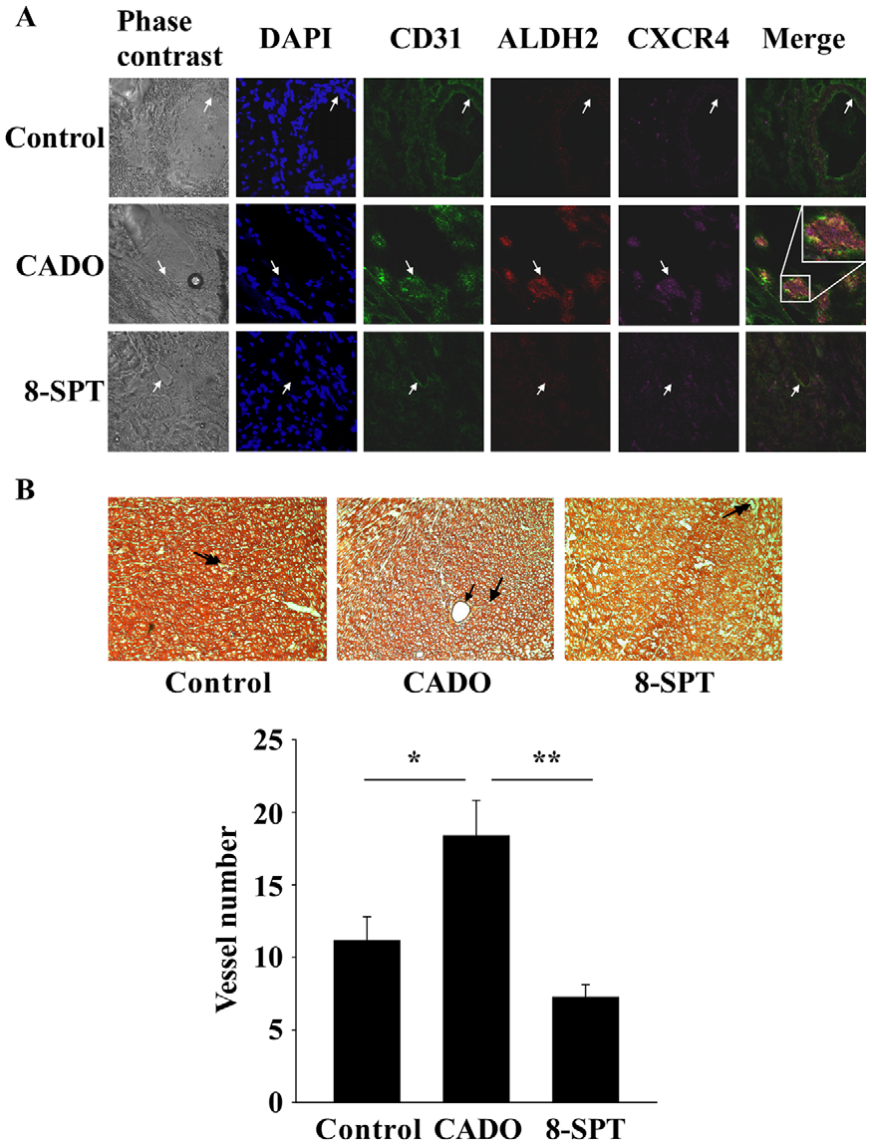
CADO treatment improves EPC recruitment to the heart and angiogenesis. LAD-occluded rats were treated with vehicle (n = 7), CADO (n = 8) or CADO +8-SPT (n = 8) as described in details in Methods section. After sacrifice, cardiac sections were performed and used for histological stainings. A. Immunostainings of CD31 (green), ALDH2 (red) and CXCR4 (pink) in cardiac sections obtained in the border zone, 2 months after MI. Nuclei are stained in blue by DAPI. Merge: overlay of CD31, ALDH2 and CXCR4 stainings. Arrows indicate blood vessel membrane. Representative pictures are shown. Magnification: ×400. B. Upper panel: representative cardiac sections 2 months after MI showing the border zone stained with hematoxylin and eosin. Arrows indicate blood vessels. Lower panel: quantitative analysis of the number of vessels in the border zone of the infarct. Magnification: ×100. Results are mean ± SEM. * P<0.05; ** P<0.001.

The effect of Ado on EPC recruitment to the heart was evaluated in rats. MI was induced in 23 rats through ligation of the LAD coronary artery. LAD-ligated rats received twice daily injections of NaCl (control group, n = 7), or the stable analog of Ado 2-Chloroadenosine (CADO, n = 8), or CADO with the non-selective antagonist of Ado receptor 8-SPT (n = 8). Treatments were given for 2 months, starting 7 days after coronary ligation. Cardiac sections were stained for CD31, CXCR4 and ALDH2. In CADO-treated rats, all 3 markers were markedly enhanced in the infarct border zone. This effect was blunted when the effect of Ado was blocked by 8-SPT (Fig. 7A).

Finally, we assessed whether this enhanced recruitment of EPC resulted in improved angiogenesis. CADO-treated rats exhibited an enhanced number of blood vessels within the border zone, indicating that EPC recruitment indeed favored revascularization of the infarct border zone (+65% when compared to controls, P = 0.03) (Fig. 7B). This angiogenic effect was prevented by 8-SPT.

Therefore, our results clearly show that Ado stimulates EPC recruitment and angiogenesis in the infarcted heart.

## Discussion

In the present study, the effect of Ado on the migration of EPC was investigated. First, we observed that Ado modulates the expression of several chemokines and chemokine receptors in EPC cultured in vitro. In particular, CXCR4 was up-regulated and this was associated with stimulation of cell migration. Then, we demonstrated that Ado improves the recruitment of EPC to the ischemic heart and this is accompanied by enhanced vascularization.

Microarrays were used to investigate the effects of Ado on EPC at a genome-wide level. Ado regulated the expression of several members of the chemokine family. Since CXCR4 was one of the most affected and is the major regulator of EPC chemotaxis after binding of SDF-1α, we focused our investigations on this receptor.

Up-regulation of CXCR4 mRNA expression by Ado was significant (3-fold increase). This up-regulation was absent in monocytes, suggesting that Ado does not induce an uncontrolled inflammatory response which could be consecutive to the recruitment of several hematopoietic cell types.

The increase of CXCR4 expression at the cell surface was modest, but highly reproducible (P<0.001). This is probably a consequence of receptor internalization. Similar increases of CXCR4 at the cell surface have been shown to be sufficient to stimulate EPC migration [28]. Therefore, we were not surprised to observe that up-regulation of CXCR4 expression by Ado stimulates the migration of EPC, both towards recombinant SDF-1α and conditioned medium from fibroblasts. Fibroblasts are a major source of SDF-1α in the heart.

In the current study, the increase of CXCR4 expression was significant at a dose of 1 µmol/L of Ado, a dose that can easily be encountered extracellularly in the ischemic heart [29]. Indeed, in conditions of ischemia, Ado can accumulate in the heart to 100 µmol/L [29]. Our results are therefore physiologically relevant.

We profiled the expression of Ado receptors on EPC, and observed significant regulation of their expression by Ado itself. Adenosine receptors appear to be tightly regulated. In inflammatory cells for instance, we and others have shown a high level of regulation following activation [14]–[16], [30], [31]. In addition to transcriptional control, Ado receptors are also regulated by internalization [32]. In EPC, Ado increased the expression of A_2_-type receptors. Mechanistic studies using pharmacological agents and RNA interference suggested that the A_2B_ receptor is responsible for the effect of Ado on CXCR4. This is consistent with the reported over-expression of CXCR4 through A_2A_ and A_2B_ receptors in human carcinoma cells [33]. Our findings suggest that drugs having high affinity for the A_2B_ receptor may have a significant effect on EPC recruitment. However, from our data, some contribution of the A_2A_ receptor on CXCR4 and EPC cannot totally be excluded. The A_2A_ receptor is involved in EPC mobilisation and angiogenesis in a wound model [34]. In contrast, knock-out mice for the A_2B_ receptor showed an over-expression of CXCR4 on leukocytes [35]. This discrepancy with our results and others' [33] may be related to species differences. Indeed, it is known that Ado receptors activity vary with species [36]. Our experiments were performed with human cells.

To validate our in vitro observations in an in vivo setting, we analysed EPC recruitment in rats subjected to MI and supplemented with CADO and/or 8-SPT. We observed an increase of CXCR4 staining in cardiac sections from CADO-treated rats. This increase was paralleled by an increase of ALDH2 staining. A high level of ALDH2 activity has been described in EPC [37]. Transplantation of bone marrow cells with high ALDH2 activity improves revascularisation of ischemic limbs and cord blood progenitors with high ALDH2 activity improve vascular density after acute MI [38], [39]. The increased expression of ALDH2 in CADO-treated rats indicates a stimulation of the recruitment of EPC with high regenerative potential. In LAD-occluded control rats, we did not observe significant ALDH2 and CXCR4 stainings, suggesting that EPC recruitment is weak in these animals. In addition, administration of 8-SPT blunted the effect of CADO. Therefore, our data show that Ado is a potent stimulus of EPC recruitment to the heart.

The recruitment of EPC to the heart of CADO-treated rats was associated with an enhanced expression of CD31, an endothelial marker, and blood vessel number. This observation is consistent with a stimulation of the revascularization of the ischemic heart by CADO. However, whether this is a direct consequence of EPC recruitment following CADO administration is unclear. Indeed, Ado is pro-angiogenic and indirect effects, through stimulation of the production of vascular endothelial growth factor by inflammatory cells for instance [16], may also be involved. Of note, EPC failed to produce vascular endothelial growth factor, even upon treatment with Ado (not shown). Tano et al. [11] showed that ischemia inhibits the expression of miR-150 in bone marrow derived mononuclear cells and activates its target gene CXCR4. We therefore investigated whether Ado could regulate CXCR4 expression through modulation of miR-150. In these experiments, EPC were cultured under ischemic conditions (i.e. serum and oxygen deprivation) to mimic cardiac stress. In parallel to an increase of CXCR4 expression, we observed a decrease of miR-150 when cells were treated with Ado. Since CXCR4 is a known target of miR-150 [11], we sought to verify this mechanism in our system. Using a luciferase gene reporter assay, we were able to show that miR-150 indeed binds CXCR4 and thereby decreases CXCR4 expression. In addition, in experiments with actinomycin D, which blocks RNA transcription, we were able to show that Ado up-regulates CXCR4 transcription. Overall, it appears that up-regulation of CXCR4 expression by Ado may be controlled by multiple pathways involving both transcription of CXCR4 and of miR-150.

Blocking endogenous miR-150 with anti-miR-150 or enhancing the pool of intracellular miR-150 by transfection of pre-miR-150 did not alter CXCR4 expression per se. Interestingly, down-regulation of miR-150 by anti-miR-150 potentiated the increase of CXCR4 expression by Ado, and, on the opposite, up-regulation of miR-150 by pre-miR-150 prevented the increase of CXCR4 expression by Ado. These data suggest that, under ischemic conditions, Ado enhances CXCR4 expression through down-regulation of miR-150. However, modulation of miR-150 expression by anti-miR-150 or pre-miR-150 did not affect cell migration. This may be related to our recent observation that EPC migration is controlled not only by CXCR4/SDF-1α, but also by CCR1/MCP-3 [40].

So far, very few reports have studied miR-150 in stem cells. miRNA profiles have identified miR-150 in EPC, human umbilical vein endothelial cells, and human coronary artery endothelial cells [41]. Our results confirmed the expression of miR-150 in EPC and suggested that this miRNA may be a potential target to enhance the regenerative capacity of these cells. However, while miR-150 appears to slow EPC migration in our study, a previous study reported that miR-150, secreted by monocytes, enhances the migration of human microvascular endothelial cells [42]. Interrogation of web-accessible databases identified over 400 predicted targets of miR-150. Additional studies are required to test whether miR-150 affects the expression of other genes regulating the functionality of EPC. Ultimately, the usefulness of miR-150 as a target for cardiac repair will have to be determined in animal models.

In conclusion, we have shown that Ado stimulates the migration of EPC. In vitro, the mechanism involves A_2B_ receptor activation, up-regulation of CXCR4 expression and down-regulation of miR-150. This effect is associated with enhanced angiogenesis in the ischemic heart. These findings suggest that A_2B_ agonists should be tested in models of cardiac repair.
